# Multi-target action of β-alanine protects cerebellar tissue from ischemic damage

**DOI:** 10.1038/s41419-022-05159-z

**Published:** 2022-08-29

**Authors:** Olga Kopach, Dmitri A. Rusakov, Sergiy Sylantyev

**Affiliations:** 1 Queen Square Institute of Neurology, University College London, London, WC1N 3BG UK; 2 Bogomoletz Institute of Physiology, Kyiv, 01024 Ukraine; 3 Rowett Institute, University of Aberdeen, Ashgrove Road West, Aberdeen, AB25 2ZD UK

**Keywords:** Cell death in the nervous system, Experimental models of disease

## Abstract

Brain ischemic stroke is among the leading causes of death and long-term disability. New treatments that alleviate brain cell damage until blood supply is restored are urgently required. The emerging focus of anti-stroke strategies has been on blood-brain-barrier permeable drugs that exhibit multiple sites of action. Here, we combine single-cell electrophysiology with live-cell imaging to find that β-Alanine (β-Ala) protects key physiological functions of brain cells that are exposed to acute stroke-mimicking conditions in ex vivo brain preparations. β-Ala exerts its neuroprotective action through several distinct pharmacological mechanisms, none of which alone could reproduce the neuroprotective effect. Since β-Ala crosses the blood-brain barrier and is part of a normal human diet, we suggest that it has a strong potential for acute stroke treatment and facilitation of recovery.

## Introduction

Brain stroke is one of the leading causes of death worldwide, with the ischemic mechanism (blood flow obstruction) generating ~87% of stroke cases [1]. There are two procedures commonly considered for blood vessel recanalization in stroke patients: thrombolysis with recombinant tissue plasminogen activator, which promotes degradation of the thrombus’ fibrin, and the instrumental removal of the thrombus (endovascular thrombectomy) [2]. A statistically significant improvement is achievable within the 4.5 h time window for thrombolysis [2] and 7.3 h for thrombectomy [3]. Therefore, the search for drugs has been focused on extending the therapeutic time window, which would increase the success rate of recanalization therapies.

Classically, ischemic damage causes three main types of cell death in neural tissue: necrosis, apoptosis, and autophagy [4–6], with the common underlying cause being glutamatergic excitotoxicity [7–9]. Oxygen-glucose deprivation (OGD), the main effect of ischemia, leads to the depletion of intracellular ATP and, consequently, loss of the ATPase activity, which supports glutamate uptake by neuronal transporters [10]. The ensuing increase in the extracellular glutamate concentration is the main factor triggering excitotoxicity through the excessive activation of excitatory glutamate receptors [11]. One strategy to counteract such excitotoxicity is to increase the activation of inhibitory receptors, such as GABAa receptors (GABAaRs). However, such hyperactivation triggers deleterious effects, such as increased neural cell death under OGD [12], impaired functional recovery after stroke [13], and it could provoke the development of absence epilepsy [14]. Another method to suppress glutamatergic excitotoxicity is the downregulation of glutamate receptors. Despite promising results obtained in this context, further clinical trials continue to repeatedly demonstrate an unacceptable level of the side effects of glutamate receptor antagonists [15–17], such as nausea, vomiting, and symptoms of psychosis [18].

The traditional approach in designing anti-stroke therapies has focused on a single pharmacological target activated with high potency. However, it has recently emerged that having simultaneous ‘subthreshold’, lower potency actions on several targets provide a superior therapeutic index if compared with selective high-efficacy drugs [19, 20]. Such a strategy is expected to be particularly effective for neurological conditions involving complex interacting mechanisms [19, 21], and its strong potential for ischemia has been predicted by modeling studies [22]. Thus, the Stroke Treatment Academic Industry Roundtable consensus suggests focusing on agents with multiple mechanisms of action while accounting for interactions between neuroprotective and thrombolytic therapies [23].

The aliphatic amino acid β-Alanine (β-Ala) has several action sites or effectors in the CNS. It has an inhibitory effect by activating glycine receptors (GlyRs) and GABAaRs while suppressing excitation by competing with glycine for co-agonist action on NMDA-type glutamate receptors (NMDARs) [24]. Additionally, β-Ala suppresses GABA transporters (GABAT) [25], thus increasing the extracellular concentration of GABA. In the systemic context, β-Ala crosses the blood-brain barrier [26, 27] through an interaction with the specific concentration-dependent transporter [28–30]: after intraperitoneal administration, it develops significant effects in the brain within 30 m [27]. These observations make β-Ala a potentially promising agent in brain ischemia treatment. Indeed, β-Ala has recently been shown in animal models to protect intestinal [31], mesenteric [32], and cardiac [33] tissue from ischemic damage. At the same time, β-Ala has had no significant side effects when consumed chronically by humans in elevated amounts [34, 35]. Therefore, in our study, we aimed to explore β-Ala as a perspective pharmacological tool increasing the therapeutic time window in anti-stroke therapy.

Here, we aimed to explore β-Ala action during OGD in the cerebellum. The cerebellum has traditionally been viewed as a regulator of motor and movement functions [36]. Over the past decades, however, a consensus has formed regarding its wider role in cognition, emotion, and autonomic function [37–41]. The current models of cerebellar function consider sensory input filtering, processing, and interpretation through a di-synaptic circuit: mossy fibers (MFs) → cerebellar granule cells (CGCs) → Purkinje cells (PCs) [42–45]. CGCs, which play the main regulatory role in this circuit, are also an established subject of studies on neural cell apoptosis, necrosis and survival [46], making these cells a suitable focus of ischemia research. In our recent work, we showed that the sensory signal handling by CGCs and PCs demonstrated in vivo [41] can be reproduced in brain slices [47], which are highly sensitive to ischemic damage [48, 49]. In this context, shifts in excitation-inhibition balance (including that in the cerebellar CGC-PC circuit) are among the most important triggers of ischemia-induced functional disorders, excitotoxicity, and cell death [11, 50, 51]. Therefore, we sought to test the protective potential of β-Ala against ischemic damage in the MF→CGC→PC circuit by using highly controlled in vitro protocols in acute cerebellar tissue.

## Results

### Neuroprotective effect of β-Ala on CGCs and PCs in ischemic conditions

**Fig. 1:**
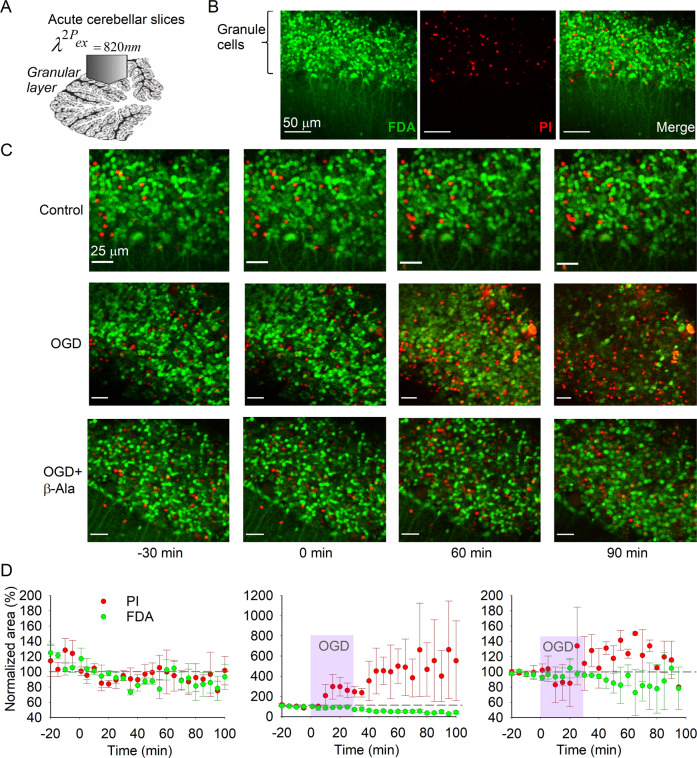
β-Ala prevents the OGD-induced CGC death. **A** An experimental arrangement for monitoring ischemic changes in neuronal viability vs. cell death in the CGC layer, using two-photon excitation (2PE) imaging in acute cerebellar slices. **B** Representative images of Z-projection in the CGC layer to show FDA labeling of the cells (green fluorescence channel, indicates viable cells), PI staining (red fluorescence channel, indicates dead cells) and merged image. Images were taken as 512 × 512 frame scans over tissue depth: total scanned 48-μm depth; *λ*^2P^_ex_ = 820 nm. **C** Examples of time-lapse 2PE images (merged FDA and PI) taken from the same region of interest in the CGC layer over the time of recording in different experimental conditions. From top to bottom: images of the CGC layer (Z-projection) in a control acute cerebellar tissue, a slice subjected to OGD (30-m duration) after 30-m control time interval followed by reperfusion, and tissue subjected to OGD in the presence of β-Ala, as denoted. Time marks at the bottom apply to all images; the “0 m” time mark denotes the start point of OGD (30-m duration). **D** Summary of image analyses for the proportion of viable (FDA fluorescence) vs. dead CGCs (PI, red fluorescence) over the time in control (*n* = 6 slices/4 animals, left plots), OGD (*n* = 8 slices/4 animals, middle) and OGD in the presence of β-Ala (*n* = 5 slices/3 animals, right plots). Data normalized to the control values (image captured 30 m before the OGD onset corresponds to 100% level). Notes apply to all three plots.

We first aimed to validate the ischemic neuronal cell death in the cerebellar tissue following OGD of 30 m duration and to find whether β-Ala protects neural tissue against ischemia-related cell death. Multiplexed two-photon excitation (2PE) imaging was performed in live cerebellar slices (Fig. 1A) to quantify neuronal cell death during 30 m OGD followed by reperfusion for typically 60–80 m. The proportion of viable (FDA-labeled) CGCs vs. dead (PI-stained) cells (Fig. 1B) were calculated before and during OGD and the following reperfusion for the entire duration of recordings (120–130 m). For the data analysis, we used a two-way repeated-measures analysis of variance with Geisser–Greenhouse’s correction for sphericity (RM-ANOVA) for the time (factor 1) and experimental conditions (control, OGD, and OGD in the presence of β-Ala – factor 2), after which Tukey post-hoc test was applied on factor 2.

In the first set of experiments, we assessed the OGD-induced cell death in the CGC layer. No significant changes in the proportion of viable CGCs were observed in control tissue in our experimental settings for ~2 h of recordings (*n* = 6 slices/4 animals); however, a robust rise in the fraction of dead CGCs accompanied by a gradual decrease in viable CGCs were observed shortly after the OGD onset that further progressed following reperfusion (*n* = 8 slices/4 animals; Fig. 1C, E). Supplementing 1 mM of β-Ala prevented these changes (*n* = 5 slices/3 animals; Fig. 1C, E). For the viable CGCs, a significant impact was caused by the time and experimental conditions (factor 1, *F*_(2.7, 25.23)_= 5.034, *p* = 0.0087; factor 2, *F*_(2, 16)_ = 9.98, *p* = 0.0015), but not by their combination (factor 1 × factor 2, *F*_(46, 215)_= 1.384, *p* = 0.066; Tukey test on factor 2: *p* < 0.0001 for control vs. OGD, *p* < 0.0001 for OGD vs. OGD with β-Ala, but *p* = 0.86 for control vs. OGD with β-Ala). For the fraction of dead CGCs, both experimental conditions and the combination of time and experimental conditions caused a significant impact (factor 2, *F*_(2, 12)_ = 6.72, *p* = 0.011; factor 1 × factor 2, *F*_(46, 157)_ = 1.56, *p* = 0.023), but not the time alone (factor 1, *F*_(1.62, 11.04)_ = 1.202, *p* = 0.33; Tukey test on factor 2: *p* < 0.0001 for control vs. OGD, *p* < 0.0001 for OGD vs. OGD with β-Ala, and *p* = 0.075 for control vs. OGD with β-Ala).

**Fig. 2:**
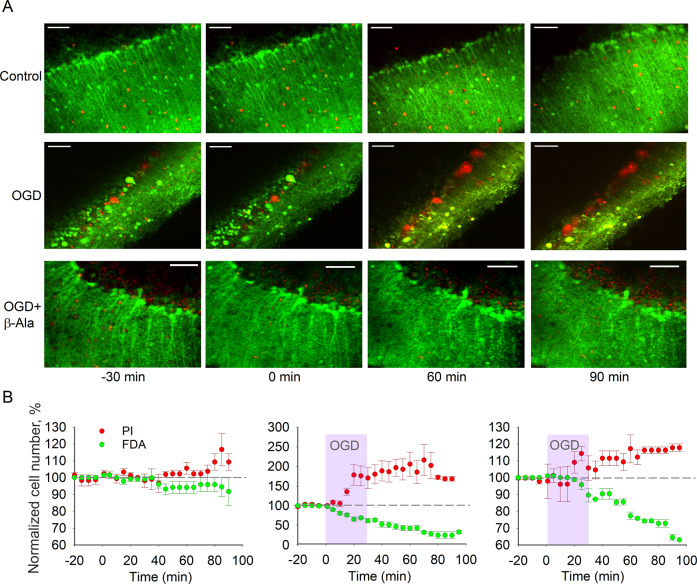
β-Ala prevents the OGD-induced death of PCs. **A** Experimental arrangement same as shown in Fig. 1A, but for the PC layer. Examples of time-lapse 2PE images of PCs in acute cerebellar tissue slice over the time of recording in different experimental conditions. Images, Z-projection of the PC layer (merged FDA and PI fluorescence channels). From top to bottom: a control acute cerebellar tissue, a slice subjected to OGD and OGD in the presence of β-Ala, as denoted. Time marks at the bottom apply to all images; “0 m” time mark denotes the start point of OGD (30-m duration). **B** Summary of image analyses for the number of viable (FDA fluorescence) vs. dead PCs (PI, red fluorescence) over the time in control (*n* = 7 slices/5 animals, left plots), OGD (30-m duration) followed by reperfusion (*n* = 8 slices/5 animals, middle) and OGD in the presence of β-Ala (*n* = 5 slices/3 animals, right plots). Data normalized to the control values (image captured 30 m before the OGD onset corresponds to 100% level). Notes apply to all three plots.

In the next set of experiments, we evaluated the OGD-induced neuronal death of PCs, with a possible effect of β-Ala on it. Similar to the observations in CGCs, β-Ala revealed its neuroprotective effect on PCs in ischemic conditions (control: *n* = 7 slices/5 animals; OGD: *n* = 8 slices/5 animals; OGD + β-Ala: *n* = 5 slices/3 animals; Fig. 2). For the amount of viable PCs, the impact of both factors and their combination was significant: factor 1, *F*_(3.85, 46.4)_ = 38.04, *p* < 0.0001; factor 2, *F*_(2, 17)_ = 93.98, *p* < 0.0001; factor 1 × factor 2, *F*_(44, 265)_= 13.25, *p* < 0.0001; Tukey test on factor 2: *p* < 0.0001 for all data set comparisons. Same was for the amount of dead PCs: factor 1, *F*_(1.07, 12.59)_ = 7.89, *p* = 0.0141; factor 2, *F*_(2, 17)_ = 12.25, *p* = 0.0005; factor 1 × factor 2, *F*_(44, 260)_ = 5.74, *p* < 0.0001; Tukey test on factor 2: *p* < 0.0001 for control vs. OGD, *p* < 0.0001 for OGD vs. OGD with β-Ala, *p* = 0.0188 for control vs. OGD with β-Ala.

Thus, β-Ala protects CGCs and PCs against ischemia-induced cell death to a similar extent between the neuronal types.

### β-Ala activates GABAaRs, GlyRs, and NMDARs

We next examined the mechanism(s) of neuroprotection produced by β-Ala in ischemia. For this, we first determined the effect of 1 mM β-Ala (the neuroprotective concentration determined above) on the activity of different receptors, in particular GABAaRs, GlyRs, and NMDARs. To counteract the impact of β-Ala on GABATs (i.e., reduced uptake of GABA from the extracellular space), we adopted the method introduced for the cerebellar tissue by others [25]: pre-loading with β-Ala. We incubated the cerebellar slices with 1 mM β-Ala for one h to allow its intracellular accumulation, hence targeting GABATs; 100 µM bicuculline was also added against GABAaR hyperactivation. The tissue was washed out before the OGD onset.

To establish the effect of β-Ala on the receptors’ activity, we used electrophysiology. To this end, we implemented recordings from the outside-out membrane patches to which we applied varied agonists using a multiple solution exchange system as described previously [52]. The experimental protocol included applications of 1 mM β-Ala and highly-selective ligands at different concentrations in paired recordings (from the same outside-out patches) to dissect the receptor-mediated activity pharmacologically (see Methods for details). We recorded the single-channel openings and calculated the open-time fraction.

**Fig. 3:**
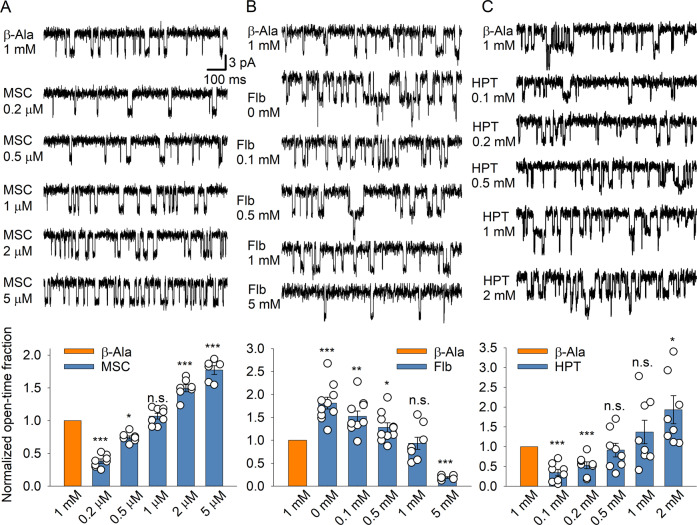
Single-channel recordings demonstrate the effect of β-Ala on GABAaRs, NMDARs, and GlyRs, with the concentration-dependent effects of the selective receptor ligands. **A** Top: recordings of the single-channel GABAaR openings elicited by 1 mM of β-Ala and different concentrations of a selective GABAaR agonist muscimol (MSC) in the same outside-out patch (*n* = 8 recordings/6 animals). Bottom: statistical summary of the GABAaR open-time fraction, normalized to the response produced by 1 mM β-Ala. MSC at the concentration of 1 µM produces the equipotent effect as that elicited by β-Ala. **B** Same as in A, but for the single-channel NMDAR openings activated by 1 mM of β-Ala and different concentrations of Felbamate (Flb). 1 mM Flb was found as the equipotent concentration to β-Ala (*n* = 10 patches/cells per paired recordings made from 7 animals). **C** Same as in A, but for the single-channel GlyR openings activated by β-Ala and different concentrations of hypotaurine (HPT). HPT at the concentrations of 0.5 mM and 1 mM produced the equipotent effect to that of β-Ala (*n* = 8 recordings/8 animals). Scale bars and vertical axis label apply to **A**–**C**. *P < 0.05, **p < 0.01, ***p < 0.001, n.s. non-significant compared to the effect of β-Ala (Student’s paired *t*-test).

For the activation of GABAaRs, we used muscimol (MSC), a selective orthosteric GABAaR agonist that does not interfere with the other action sites of β-Ala [53]. Our data showed that 1 mM β-Ala supported an open-time fraction that was statistically indistinguishable from that generated by 1 µM MSC (*n* = 8 paired recordings, 6 animals; Fig. 3A).

Next, we quantified the effect of β-Ala on NMDARs and compared it with that elicited by different concentrations of the NMDAR antagonist felbamate (Flb), which shares a glycine binding site with β-Ala [54]. Since varied concentrations of glycine (Gly) were reported in the extracellular space of the cerebellum: from 7.6 ± 2.1 µM [55] and 18.3 ± 9.2 µM [56] to 27.7 ± 12.1 µM [57], we used in our experiments 10 µM Gly in combination with glutamate (Glu) for the activation of NMDARs. Similarly, different concentrations of Glu were reported in the cerebellum: 5.1 ± 0.3 µM [55] and 13 ± 7.5 µM [57]. In our experimental settings, we applied 10 µM Glu in Mg^2+^-free solution together with β-Ala or Flb. The results showed that the NMDAR open-time fractions elicited by 1 mM β-Ala were statistically similar to that activated by 1 mM Flb (*n* = 10; 7 animals total; Fig. 3B).

Similarly, we determined the effect of β-Ala on GlyRs. We compared it with the effects of different concentrations of the selective GlyR ligand hypotaurine (HPT), which shares its binding site with β-Ala [58]. The single-channel GlyR open-time fraction elicited by 1 mM Ala was statistically indistinguishable from that elicited by two concentrations of HPT: 0.5 mM and 1 mM (*n* = 8 from 8 animals; Fig. 3C).

Together these data demonstrate the effects of 1 mM β-Ala on different receptors, which can be reproduced by 1 µM MSC for GABAaRs, 1 mM Flb for NMDARs, and 1 mM HPT for GlyRs.

### β-Ala alleviates the OGD-induced anoxic depolarization in CGCs and PCs

Having established the effect of β-Ala on GABAaRs, GlyRs, and NMDARs, we aimed to next determine the role of each of those receptors in ischemic neuronal dysfunction. Since anoxic depolarization (AD) is a hallmark of ischemic brain damage [48, 59], we performed the measurements of the OGD-induced AD in CGCs and PCs using whole-cell recordings.

**Fig. 4:**
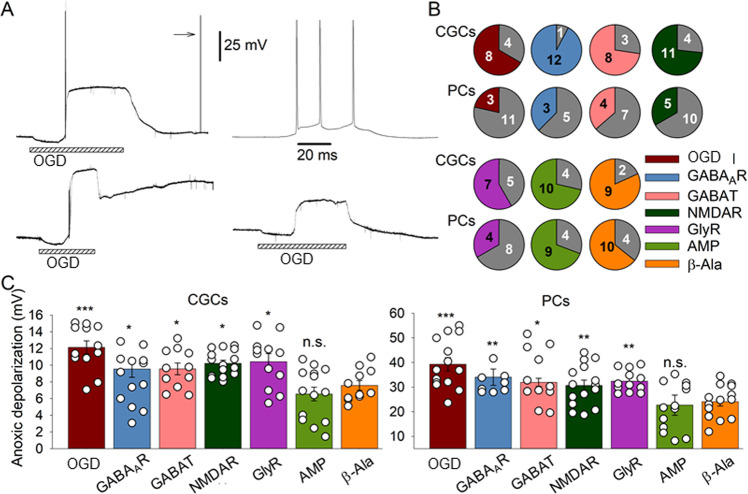
β-Ala alleviates the OGD-induced anoxic depolarization (AD) in CGCs and PCs by simultaneously targeting GABAaRs, NMDARs, GlyRs, and GABA transporters. **A** Examples of the OGD-induced AD recordings. Top: a cell recovered its membrane potential after an OGD episode back to a control level. Note spontaneous APs of typical kinetic profile evoked post-OGD (arrow mark) to confirm functional recovery. Top right: recorded spontaneous APs shown at an expanded time scale. Bottom left: a cell failed to restore its membrane potential after the OGD-induced AD. Bottom right: in the presence of β-Ala, a cell displayed a lower AD amplitude and recovered its membrane potential post-OGD. Bars at the bottom mark a 15-m OGD episode, 25 mV scale bar apply to all traces. **B** Summary of the proportion of CGCs and PCs that recovered their membrane potential after the OGD-induced AD or not recovered (gray color). The number of cells indicated (total *n* = 8–15 for each test, 10–13 animals/group). Charts are color-coded for the selective activation of different receptors or β-Ala as denoted. AMP (β-Ala mimicking protocol) includes simultaneous activation of GABAaRs, NMDARs, GlyRs, and GABA transporters using the concentrations of selective ligands equipotent to the β-Ala effect. **C** Statistical summary of the OGD-induced AD amplitude in CGCs (left plots) and PCs (right plots) in different experimental conditions (color-coded): in the presence of β-Ala or selective receptor agonists used separately or simultaneously. *P < 0.05, **p < 0.01, ***p < 0.001, n.s. non-significant compared to the effect of β-Ala (Student’s *t*-test); total *n* = 8–15 cells/8–11 animals tested per group.

For the quantification of AD, two parameters were used: (i) the AD amplitude and (ii) the proportion of cells that restored membrane potential to the level “Control ± 5 mV” for PCs and “Control ± 2 mV” for CGCs (termed thereafter ‘recovered cells’). We found that β-Ala increased the proportion of recovered PCs (3 vs. 11 cells in OGD, 13 animals; and 10 vs. 4 cells in OGD with β-Ala, 10 animals, respectively; *p* = 0.023, Fisher’s exact test), but not CGCs (8 vs. 4 cells in OGD, and 9 vs. 2 cells in OGD with β-Ala, *p* > 0.999, Fisher’s exact test; 10 animals in both cases; Fig. 4B). Also, β-Ala significantly decreased the AD amplitude in PCs: 39.25 ± 2.71 mV in OGD (*n* = 14 from 9 animals) vs. 24.1 ± 1.77 mV in OGD with β-Ala (*n* = 14 from 11 animals, *p* < 0.001, Student’s *t*-test; Fig. 4C). The decrease in the AD amplitude was also observed in CGCs: 12.12 ± 0.79 mV in OGD (*n* = 12 form 8 animals) vs. 7.56 ± 0.58 mV in OGD with β-Ala (*n* = 11 from 8 animals, *p* < 0.001, Student’s *t*-test; Fig. 4C).

Activation of GABAaRs, GlyRs, NMDARs, and GABAT separately using the selective agonists at the concentrations equipotent to β-Ala did not increase the proportion of recovered cells (*n* = 8–15 per case, *p* > 0.16 compared to control, Fisher’s exact test; Fig. 4B). For the AD amplitude, activation of each of the receptors or GABAT resulted in a significant decrease in the AD amplitude, in both PCs and CGCs (*n* = 8–15, *p* < 0.05 per case, Student’s *t*-test); however, not to the same extent as β-Ala did (Fig. 4C).

Finally, we probed the protocol to activate all four targets simultaneously – GABAaRs, GlyRs, NMDARs, and GABAT – to reproduce the effect of β-Ala. In this experimental protocol, named thereafter “β-Alanine mimicking protocol” (AMP), we applied a mixture of 1 µM MSC, 1 mM Flb, and 1 mM HPT to the tissue pre-incubated with β-Ala (to inhibit GABAT), followed by its subsequent washing out. This protocol produced a decrease in the AD amplitude to a similar extent as β-Ala did (Fig. 4C). In CGCs, the amplitude was: 6.54 ± 0.83 mV for AMP (*n* = 14 from 10 animals) vs. 7.56 ± 0.58 mV for β-Ala (*n* = 11 from 6 animals, *p* = 0.326); in PCs: 21.84 ± 2.62 mV (*n* = 13 from 9 animals) vs. 24.1 ± 1.77 (*n* = 14 from 9 animals, *p* = 0.481, Student’s *t*-test), respectively. Also, it enabled replicating the effect of β-Ala on the proportion of recovered PCs (9 vs. 4, in 9 animals *p* = 0.021, Fisher’s exact test) but not CGCs (*p* = 0.999, Fisher’s exact test, 9 animals).

These results demonstrate the multifaceted mechanism of neuroprotection produced by β-Ala, by dampening neuronal hyperexcitability and recovering membrane potential in ischemic conditions through activation of GABAaRs, GlyRs, NMDARs, and GABAT. The effect of β-Ala is significantly higher than that produced by activating any of the above targets separately. A contribution of other possible targets seems unlikely given the equal effects produced by β-Ala and AMP. Altogether it makes β-Ala a promising agent for further research focus.

### β-Ala impedes the ischemic impairments in CGC-PC circuitry

The primary role of the CGC layer is filtering sensory inputs and transferring patterns of action potentials (APs) to PCs [41]. Having established the β-Ala effects on the OGD-related hyperexcitability of CGCs and PCs, we next studied functional connectivity in the CGC→PC circuitry.

**Fig. 5:**
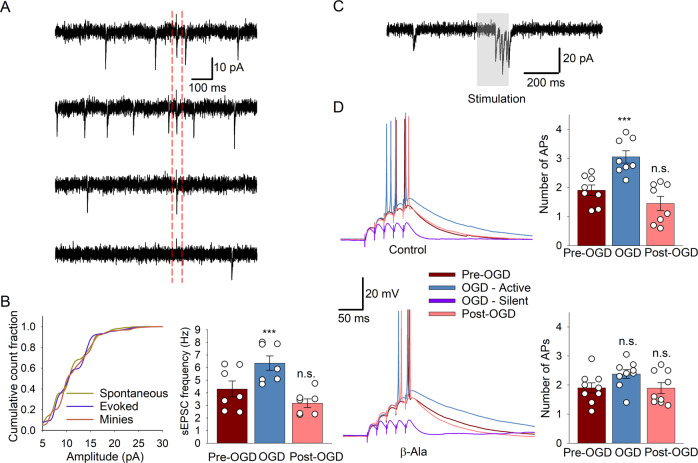
β-Ala prevents the ischemic disruption of the CGC activity. **A** Example whole-cell recordings from a CGC in response to stimulation of a single mossy fiber (MF), mimicking sensory inputs to CGCs. From top to bottom: control, OGD (3 m after ischemic onset), reperfusion, TTX (1 µM) added to inhibit synaptic transmission. Dashed red lines indicate EPSCs evoked by stimulation of a MF. **B** Left, statistical summary for the recordings of spontaneous EPSCs, EPSCs evoked by MF stimulation (as shown in **A**), and mini EPSCs, plotted as normalized cumulative amplitude histograms. Note the similar histograms between different currents, indicating that sensory inputs induce similar responses (generated by similar neurotransmitter release). Right: statistical summary of the spontaneous EPSCs shows the increased frequency upon OGD (5 m after OGD onset). ***P < 0.001, n.s. non-significant compared to control (Student’s paired *t*-test), total *n* = 7 cells/slices from 6 animals. **C** Representative voltage-clamp recording from a CGC showing the currents generated in response to a burst stimulation (5 stimuli applied) of a single MF. **D** Examples of current-clamp recordings from individual CGCs in response to a burst stimulation of a single MF (5 stimuli) in control, OGD and post-OGD without (top) and in the presence of β-Ala (bottom). Scale bars and color codes apply to both cases. Plots, summary of the number of APs per individual CGCs in response to MF stimulation (5 stimuli). ***P < 0.001, n.s. non-significant compared to before OGD (Student’s paired *t*-test), *n* = 9 cells/slices from 6 animals.

To reproduce sensory inputs in CGCs, we implemented an experimental protocol as in our earlier work [47]. In particular, individual CGCs were recorded for evoked APs in response to stimulation of a single MF in the cerebellar white matter (see Methods for details). We first confirmed a high similarity of mini excitatory post-synaptic currents (EPSCs), spontaneous EPSCs, and evoked EPSCs while stimulating MF (Fig. 5A, B). This resembles sensory inputs to CGCs in vivo [41, 47]. Then, we recorded APs in response to burst stimulation (5 stimuli applied at 25 Hz, in 30-s intervals between trials). Our data showed that the frequency of either spontaneous EPSCs (*n* = 7 cells/slices; Fig. 5B) or evoked APs (*n* = 8 cells/slices; Fig. 5D) increased within 5*–*7 m after the onset of OGD (active stage), and cells stopped generating APs in response to stimuli following that time period (silent stage). Therefore, we quantified AP patterns as the average number of APs per stimulation/experimental conditions (i.e. before OGD, 5 m of OGD, post-OGD), without and in the presence of β-Ala. Our results showed a significant increase in the number of APs upon OGD: from 1.91 ± 0.18 before OGD to 3.05 ± 0.21 at 5 m of OGD (*n* = 8 cells/slices from 6 animals, *p* = 0.0004, Student’s paired *t*-test). However, β-Ala eliminated the OGD-induced increase in the AP number: 1.9 ± 0.17 before vs. 2.37 ± 0.15 at 5 m after OGD with β-Ala (*n* = 9 cells/slices from 6 animals, *p* = 0.08, Student’s paired *t*-test).

**Fig. 6:**
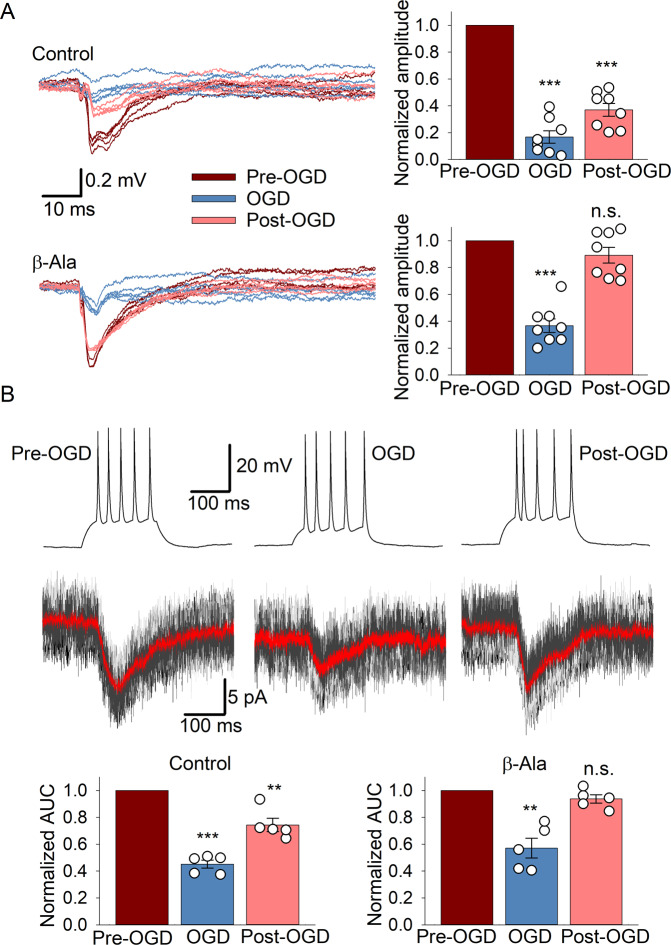
β-Ala protects the CGC-PC signaling in ischemic conditions. **A** Left, example recordings of field potentials (FPs) in the cerebellar molecular layer evoked by the CGC layer stimulation before OGD, upon OGD, and post-OGD (5 trials shown for each condition) after the deduction of stimulation artifacts (see Methods for detail). Note the recovered FPs in the presence of β-Ala (bottom recording). Right, statistical summary of the normalized FP amplitude in different experimental conditions and in the presence of β-Ala (lower plots). **P < 0.01, ***p < 0.001, n.s. non-significant (Student’s paired *t*-test), *n* = 8 slices/5 animals. **B** Examples of double-patch recordings made from CGCs (in current-clamp to elicit AP generation, shown of the top) and synaptically connected PCs (to record membrane currents in response to AP generated by a paired CGC, lower traces) in control (before OGD), upon OGD, and post-OGD. Gray traces show trials of current recordings, which were averaged – red trace. Plots, summary of the current recordings in PCs analyzed as the area under the curve (AUC) in different experimental conditions. Note the recovered current in PCs after OGD in the presence of β-Ala (right plots). **P < 0.01, ***p < 0.001, n.s. non-significant (Student’s paired *t*-test), *n* = 5 cells/slices from 5 animals.

After being generated by CGCs, excitatory signal bursts are delivered to PCs via parallel fibers in the molecular layer of the cerebellar cortex. To examine ischemic-related impairments in the CGC→PC circuitry, we carried out recordings of field potentials (FPs) in the cerebellar molecular layer triggered by stimulation of the CGC layer. Both amplitude and the time interval of FPs were calculated before OGD, upon OGD, and post-OGD. Our data revealed a significant drop in the FP amplitude upon OGD that did not recover to a control level following reperfusion (Fig. 6A). The amplitude, relative to control, was 0.167 ± 0.046 upon OGD and 0.37 ± 0.048 post-OGD (*n* = 8 slices/4 animals, *p* < 0.0001 compared to control, Student’s paired *t*-test). In the meanwhile, β-Ala improved recovery of the FP amplitude following OGD to its control level: 0.367 ± 0.051 upon OGD (*p* < 0.0001) and 0.891 ± 0.059 post-OGD (*n* = 8 slices/5 animals, *p* = 0.11 compared to that before OGD, Student’s paired *t*-test).

Finally, we investigated ischemic impairments in the signal transfer onto PCs in the CGC→PC pathway and whether β-Ala can alleviate them. For this, we carried out paired whole-cell recordings, with one CGC held in current-clamp to trigger APs while recording membrane currents in a PC in response to evoked APs within a pair of synaptically connected cells [60, 61]. The electrophysiological protocol resembling sensory signaling in the CGC→ PC circuitry was as above: 5 APs at ~25 Hz, evoked in a CGC by injecting a 200-ms depolarizing current (Fig. 6B). The responses generated in a PC by stimulating its CGC pair were averaged and analyzed as area under the response curve (AUC, Fig. 6B). Our recordings demonstrated that OGD significantly decreased the AP-induced currents in PCs: the AUC, relative to control, was 0.45 ± 0.03 (*n* = 5 cells/slices, *p* < 0.0001) upon OGD and 0.74 ± 0.05 (*n* = 5 cells/slices, *p* = 0.007 Student’s paired *t*-test) after OGD. Notably, β-Ala rescued the OGD-induced drop in the current: the AUC was 0.57 ± 0.07 (*n* = 5 cells/slices, *p* = 0.004) upon OGD but 0.94 ± 0.03 (*n* = 5 cells/slices, *p* = 0.11 compared to control, Student’s paired *t*-test, 5 animals in each case) after OGD (Fig. 6B).

## Discussion

In this study, we have tested the therapeutic potential of β-Ala as a perspective anti-ischemic brain treatment. Our data in acute ex vivo preparations provide evidence for the neuroprotective effect of β-Ala against ischemic damage that interrupts a key signaling circuitry in the cerebellum: MFs → CGCs → parallel fibers→PCs. In ischemic conditions, the disruptions found in the signal transfer within this circuitry were significantly alleviated by β-Ala. Our results suggest a neuroprotective effect on the ischemic neuronal cell death (Figs. 1 and 2), neuronal hyperexcitability (e.g., AD), impaired membrane potential maintenance (Figs. 4 and 5), and dysfunctions at the network level (Fig. 6).

Although successful drug treatment of ischemia has been reported in numerous animal studies, the observed benefits have often failed to replicate in clinical trials. This prompted a growing criticism of animal models as a reliable subject to test therapeutic strategies targeting ischemia in humans. In addition, ischemic brain damage is known to vary widely among individuals. Nonetheless, the basic cellular mechanisms of ischemic neuronal death— such as glutamate excitotoxicity, oxidative stress, lipid peroxidation, inflammation—have long been considered common across humans and other vertebrate species [62]. Since there has been no practical alternative to animal models to explore cellular mechanisms of ischemic neuronal death, acute brain tissue has remained an important tool for examining at least the key constituents of dysfunction associated with ischemic damage.

Here, we have focused on an acute OGD-model of ischemic neuronal damage using ex vivo tissue of the cerebellum, a brain region heavily involved in motor function. Indeed, signaling disruption in the CGC layer can cause sensorimotor dysfunction, which is a common hallmark of ischemia. CGCs generate AP bursts in response to sensory-evoked inputs from MFs, whereas CGC patterns of activity represent a neurophysiological mechanism to store and process sensorimotor information [42–45]. Restored by β-Ala application, the patterns of APs in CGCs (Fig. 5), which resemble sensory signaling in vivo [41, 47], suggest a neuroprotective effect regarding the cerebellar sensorimotor function prone to ischemic damage.

The present electrophysiological data point to a multifaceted mechanism underpinning the neuroprotective effect of β-Ala in the cerebellum. This mechanism includes cumulative targeting by β-Ala of several receptor types, particularly GABAaRs, GlyRs, NMDARs, and GABA transporters (demonstrated by single-channel receptor current recordings in outside-out membrane patches, Fig. 3). Our results suggest that the effect of β-Ala is unlikely due to a single mechanism of action but instead engages all the four receptor targets mentioned above. A significant contribution from other receptor types or regulatory mechanisms to the effect of β-Ala seems unlikely: simultaneous activation of all four targets by their specific ligands reproduced the β-Ala effect (Fig. 4).

An important mechanism of ischemic damage in the nervous tissue is the loss of Cl^−^ homeostasis, leading to neuronal cell death [63]. It has been found that hyperactivation of GABAaRs could effectively prevent excessive accumulation of Cl^−^ ions after ischemic episodes and thus partly restore neuronal signaling [64]. Therefore, the effect of β-Ala observed here against OGD-related excitotoxicity could be due to hyperactivated GABAaRs: our electrophysiological recordings by the β-Ala-mediated increase in the single-channel GABAaR openings lend support to this hypothesis. The concomitant activation of GlyRs by β-Ala, as evidenced by our single-channel GlyR recordings, helps prevent pathological Cl^−^ exchange and, therefore, could further guard against the negative impact of Cl^−^ disbalance following ischemia.

Importantly, β-Ala showed a neuroprotective effect in two different neuronal types, CGCs, and PCs. Inducing a neuroprotection mechanism to delay or reduce neuronal cell death in conditions of ischemia, as studied here, is among the key therapeutic priorities in the treatment of ischemia as it extends the time window during which further therapeutic intervention may be successful. The potential of β-Ala to actually reverse the cell damage remains an important and intriguing question that deserves a separate study.

Regarding the optimization of pharmacological approaches, the current discussion compares multiple, highly specific compounds (polypharmacy) with a single drug acting via multiple sites/mechanisms (polypharmacology). While there are pros and cons for either, the critical issue in using several drugs simultaneously arises from their different pharmacokinetics engaging distinct metabolic pathways, hence the chances of mutual side effects are high [65]. Therefore, the polypharmacological approach is emerging as more attractive in drug design research over the last decade [20], including therapeutic strategies against brain ischemia [66, 67]. Our study provides a new line of evidence for such an approach represented by β-Ala, and possibly its derivatives, which act simultaneously on GABAaRs, GlyRs, NMDARs, and GABA transporters.

The largest source of β-Ala in mammals is a dipeptide carnosine, from which β-Ala is released due to the activity of carnosinase enzymes [68]. Notably, a dramatic reduction in the activity of carnosinases has been found in the brain after ischemic stroke [69]. This further argues for the role of β-Ala in neuroprotective action against ischemic damage. As a native compound, β-Ala is present in a wide range of regular human diet products [35]. Importantly, there have been no adverse effects of β-Ala in humans when it was consumed in elevated amounts [34, 35]. Although little is known about the actual concentration of β-alanine in the brain, recent studies in healthy humans report a blood level of β-alanine rising up to 0.5 mM after a single ingestion of 1.4 g, within 1*–*2 h, steadily dropping many times within 5*–*6 h [70]. Thus, in principle, one could anticipate a relatively fast route for β-Ala into clinical trials. Nonetheless, further studies are urgently needed to validate the neuroprotective effects of β-Ala in vivo, both in the context of pre-treatment—to prevent the deleterious consequences of ischemic conditions in the brain – and post-treatment, to extend the time window for implementing subsequent clinical therapies.

## Materials and methods

### Animals and cerebellar slice preparation

All animal procedures were conducted in accordance with the European Commission Directive (86/609/ EEC) and the United Kingdom Home Office (Scientific Procedures) Act of 1986. Male Sprague Dawley rats were kept under standard housing conditions. Animals (3*–*5 weeks old) were sacrificed using an overdose of isoflurane. Following decapitation, the cerebellum was rapidly removed, and parasagittal slices (250–350 µm thick) were prepared on a Leica VT1200S vibratome. The slicing was performed in an ice-cold slicing solution that contained (in mM) 64 NaCl, 2.5 KCl, 1.25 NaH_2_PO_4_, 0.5 CaCl_2_, 7 MgCl_2_, 25 NaHCO_3_, 10 d-glucose and 120 sucrose, saturated with 95% O_2_ and 5% CO_2_. Once cut, slices were transferred into a bicarbonate-buffered Ringer solution containing (in mM) 126 NaCl, 3 KCl, 1.25 NaH_2_PO_4_, 2 MgSO_4_, 2 CaCl_2_, 26 NaHCO_3_, 10 d-glucose continuously bubbled with 95% O_2_ and 5% CO_2_ (pH 7.4; 300–310 mOsmol). Alternatively, slices were transferred into a solution that contained 124 NaCl, 3 KCl, 1 CaCl_2_, 3 MgCl_2_, 26 NaHCO_3_, 1.25 NaH_2_PO_4_, 10 d-glucose, continuously bubbled with 95% O_2_ and 5% CO_2_. Slices were allowed to rest for at least 60 min at 32–34 °C before the recordings started.

### Oxygen-glucose deprivation (OGD)

For the induction of ischemic neuronal cell death, OGD was used with a similar protocol as described earlier [71, 72]. Briefly, acute cerebellum slices were exposed to OGD (15 or 30-m duration) by administering a tissue sample into a Ringer solution containing 2 mM glucose and 8 mM sucrose (instead of 10 mM glucose) equilibrated with 95% N_2_ and 5% CO_2_. After the termination of OGD, the tissue was superfused with a normal Ringer solution. Acute cerebellum slices obtained from the same animals and subjected to similar procedures and the same time of experimenting but in a normal and oxygenated Ringer solution were used as control. Tissue samples were randomly allocated to experimental groups, and all samples tested were included in the analysis. No pre-determine sample-size calculation was performed; all sample sizes were based on the statistically significant difference between experimental groups.

### Electrophysiology

The MF-CGC pathway morphology is identical across the whole cerebellum, hence any part of the CGC layer with adjacent white matter can be used as a representative experimental object. Nevertheless, to preserve the MF-CGC pathway in an acute tissue slice where lobules can bend and separate into small pieces [73], slices of the cerebellar vermis rather than cerebellar hemispheres were used for electrophysiological experiments.

#### Whole-cell recordings

In our preparations, MFs were stimulated using a bipolar tungsten electrode, which was placed in the cerebellar white matter near the gyrus crest to stimulate MFs entering the granule cells layer [73]. To stimulate a single fiber, we used a θ-glass electrode pulled to ~5 μm tip diameter, filled with a perfusion solution, with wires in both channels connected to the stimulus isolator. After a CGC was patched, the position of the stimulating electrode was probed until excitatory input was evoked in a patched cell [47]. The intracellular pipette solution for voltage-clamp recordings contained (mM) 117.5 Cs-gluconate, 17.5 CsCl, 10 KOH-HEPES, 10 BAPTA, 8 NaCl, 5 QX-314, 2 Mg-ATP, 0.3 GTP; for current-clamp recordings: 126 K-gluconate, 4 NaCl, 5 HEPES, 15 glucose, 1 MgSO_4_·7H_2_O, 2 BAPTA, 3 Mg-ATP (295*–*310 mOsm, pH = 7.2); pipette resistance was 7*–*9 MΩ. Recordings were performed at 33*–*35 °C, using Multiclamp 700B amplifier; signals were pre-filtered and digitized at 10 kHz.

#### Outside-out patch recordings

To measure the single-channel openings, outside-out membrane patch recordings were carried out. For the GlyRs, the recordings were made in the presence of a cocktail of different ligands that contained 50 μM APV, 20 μM NBQX, 50 nM CGP-55845, 200 μM S-MCPG, 10 μM MDL-72222, and 1 mM pentylenetetrazole (PTZ), a GABAaR antagonist which has no or low impact on GlyR [74, 75]. To isolate the GABAaR-mediated conductance, the recordings were made in a cocktail containing 50 μM APV, 20 μM NBQX, 50 nM CGP-55845, 200 μM S-MCPG, 10 μM MDL-72222, and 1 µM strychnine. The NMDARs were pharmacologically isolated in Mg^2+^-free perfusion solution containing a mixture of 20 μM NBQX, 50 nM CGP-55845, 200 μM S-MCPG, 10 μM MDL-72222, 50 µM picrotoxin, and 1 µM strychnine. For the exchange of solutions at membrane patches we adapted a RASE protocol [52] that allowed to apply different solutions at the same patch with up to 150*–*200 μs time resolution. Briefly, membrane patches were exposed to the solution flow from a θ-glass pipette mounted on a piezo-actuator, with up to three different solutions exchanged in each application pipette channel.

#### Extracellular recording

Recordings of field potentials (FPs) were performed with a glass electrode filled with a perfusion solution; stimuli were delivered with a tungsten bipolar electrode connected to a DSA-2 stimulus isolator (Digitimer Ltd). Because local stimulation within the MF-CGC pathway generates a substantial stimulation artifact that can cover up to 50*–*90% of the evoked response, hence eliminating measurements of the FP amplitude accurately, we carried out procedure of artifact deduction at the end of each experiment. For this, we inhibited synaptic transmission with 1 µM tetrodotoxin and 10 nM ω-conotoxin MVIIC and then recorded the artifact profile (20*–*30 subsequent trials). The averaged stimulation artifact was subtracted from the FP responses for each individual recording.

### Electrophysiological data analysis

We used a total of 86 animals (*n* = 169 acute slices) for electrophysiological recordings. Voltage-clamp recordings were analyzed for the transmembrane current over time window (charge transfer), calculated as an area under the curve (AUC) of the response with the baseline current (average over ~50 ms prior to the response onset) subtracted.

For the outside-out membrane patch recordings, we quantified an open-time fraction of the single-channels. This was calculated as a ratio *t*_*o*_*/t*_*f*_, where *t*_*f*_ is a full time of recording and *t*_*o*_ is a time when the receptor(s) were in the open state. *t*_*o*_ was counted as a sum of openings with a minimum opening time of 0.2 ms, using the threshold-detection algorithm of Clampfit 10x software. Although it is virtually impossible to determine the number of channels in a membrane patch, the majority of channel openings in our recordings were single-level events. In the case there were multiple levels of channel openings in a given sweep, the opening level observed for the longest time was taken for the calculation.

### Two-photon excitation (2PE) imaging

Acute slices were bolus loaded with fluorescent probes by incubation in a Ringer solution at 32*–*35 °C, typically for 30*–*45 m. A cell-permeable tracer fluorescein-diacetate-5(6)-isothiocyanate (FDA, 20 μM) was used to visualize viable cells, and a membrane-impermeable DNA binding dye propidium iodide (PI, 10*–*20 μM) was used to label the dead cell fraction. After loading the dyes, slices were washed out and transferred to a recording chamber mounted on the stage of an Olympus BX51WI upright microscope (Olympus, Japan) equipped with an XLPlanN 25 × 1.05 objective coupled to an infrared DIC imaging system. 2PE imaging was carried out using an Olympus FV10MP imaging system optically linked to a Ti:Sapphire MaiTai femtosecond-pulse laser (SpectraPhysics-Newport). 2PE imaging was performed at *λ*_*x*_^2P^ = 820 nm, with appropriate emission filters (green channel: FDA; red channel: PI). Images were collected in image frame mode (512 × 512 pixels), at a nominal resolution of ~0.4–0.5 µm/pixel, using various digital zooms. Images were acquired as Z-stacks, typically consisting of 30–60 optical sections, collected at 1-µm steps. For the time-dependent changes in both FDA and PI-fluorescent signals, time-lapse recordings of the region of interest were made every 5 m before the induction of OGD (for ~30 m), upon OGD (30-m duration), and following reperfusion (at least, for 1 h). To avoid phototoxic damage to the tissue and to minimize bleaching, images were acquired using a laser power at its reasonable minimum (below 3–4 mW).

### Image analysis

For the quantification of time-dependent changes in cells’ viability, the proportions of viable vs. damaged (dead) cells were counted using ImageJ software (NIH, Bethesda, USA). All time-lapse images were aligned to ensure that their optical sections match each other over the entire time sequence; the projection images were processed (in 2D mode) by setting the threshold level for the green (FDA fluorescence) and red (PI fluorescence) channels separately, using the same threshold for all images across the time-lapse sequence. Both FDA- and PI-fluorescent signals were counted using an ImageJ plugin for particles/area analysis. Quantification was performed as a percentage of the PI-positive area (DNA staining—dead cells) and FDA staining (viable cells) relative to baseline (i.e., 30 m before the induction of OGD). The number of animals used for live-cell imaging experiments was 9 animals total (*n* = 27 slices tested).

### Statistical analysis

All datasets were tested for the distribution normality and homogeneity of variance (data sphericity) using the Prizm software package. Data are presented as mean ± s.e.m. if distributed normally. The Greenhouse-Geisser correction for the data sphericity violation was applied where necessary, resulting in non-integer degrees of freedom. In the case of non-normal data distribution, the non-parametric Fisher’s exact test was used. Two-way repeated-measures analysis of variance with Geisser–Greenhouse’s correction for sphericity (RM-ANOVA) followed by Tukey post-hoc test and Fisher’s exact test were performed where appropriate, using Prizm 8 software. Student’s two-tailed paired and unpaired tests were performed with MS Excel. *P*-value of ≤0.05 was taken as a threshold of significance in all tests.

### Chemicals

APV, NBQX, CGP-55845, S-MCPG, MDL-72222, picrotoxin, glycine, muscimol, hypotaurine, β-Alanine, QX-314, GABA, pentylenetetrazole, tetrodotoxin, ω-conotoxin MVIIC, glutamate, and felbamate were purchased from Tocris Bioscience; propidium iodide was purchased from Cambridge Bioscience; all other chemicals were purchased from Sigma-Aldrich.

### Reporting summary

Further information on research design is available in the Nature Research Reporting Summary linked to this article.

## Supplementary information

Reporting Summary

## Data Availability

The datasets generated and analyzed during this study are included in this article.
